# Drug-coated balloons in complex large-vessel coronary artery disease: a comprehensive review of current evidence and future perspectives

**DOI:** 10.3389/fcvm.2026.1731952

**Published:** 2026-02-24

**Authors:** Mauro Gitto, Alessandro Gabrielli, Pier Pasquale Leone, Jorge Sanz-Sanchez, Francesco Tartaglia, Valentina Bernardini, Damiano Regazzoli, Antonio Mangieri, Bernhard Reimers, Azeem Latib, Giulio G. Stefanini, Antonio Colombo

**Affiliations:** 1 Humanitas Research Hospital IRCCS, Rozzano – Milan, Italy; 2 Department of Biomedical Sciences, Humanitas University, Pieve Emanuele – Milan, Italy; 3 Montefiore Einstein Center for Heart and Vascular Care, Montefiore Medical Center, Albert Einstein College of Medicine, Bronx, NY, United States; 4 Hospital Universitari I Politecnic La Fe, Valencia, Spain; 5 Centro de Investigación Biomedica en Red (CIBERCV), Madrid, Spain; 6 EMO-GVM Centro Cuore Columbus, Milan, Italy

**Keywords:** bifurcation, chronic total occlusion (CTO), complex PCI, *de novo* coronary artery disease, drug-coated balloon (DCB), high bleeding risk, long coronary artery lesions, percutaneous coronary intervention

## Abstract

Drug-coated balloons (DCBs) are emerging as a valuable alternative to drug-eluting stents (DES) in percutaneous coronary intervention (PCI), especially in the context of complex coronary artery disease (CAD). While DES remain the standard of care in PCI, their use is associated with several well-recognized limitations, including impairment of vascular physiology, inhibition of positive remodeling, and a persistent risk, estimated at approximately 2% per year, of stent-related adverse events, which increases with increasing stent length and anatomical and clinical complexity. DCBs deliver antiproliferative agents without leaving a permanent metallic scaffold, offering the potential to reduce stent burden, preserve native vessel physiology, and shorten the duration of dual antiplatelet therapy. Their efficacy is well established in the treatment of in-stent restenosis (ISR) and *de novo* lesions in small vessels (SVD). However, the use of DCBs in large-vessel and complex lesions (such as bifurcations, long lesions, and chronic total occlusions) remains under investigation. Preliminary observational data suggest feasibility and potential benefits, particularly in carefully selected cases with adequate lesion preparation. This review synthesizes current pathophysiological insights, procedural considerations, and clinical data on the use of DCBs in complex large-vessel CAD and underscores the need for large-scale randomized trials to define their long-term safety and efficacy in this setting.

## 1 Introduction

Over the past four decades, the field of percutaneous coronary intervention (PCI) has experienced a remarkable evolution, transforming the landscape of coronary artery disease (CAD) treatment (1, 2). Early interventions using simple balloon angioplasty were limited by high rates of elastic recoil, acute vessel closure, and negative remodeling (3). The subsequent introduction of bare metal stents (BMS) addressed some of these issues by providing mechanical scaffolding, yet early in-stent restenosis (ISR) and stent thrombosis remained frequent complications (3). The development of drug-eluting stents (DES), particularly second-generation devices combined with dual antiplatelet therapy (DAPT), marked a pivotal advancement by significantly reducing the incidence of ISR and improving long-term patency rates. However, despite these technological strides, several critical limitations of DES persist, particularly in complex anatomical or clinical scenarios (4). These include impairment of native vessel physiology, delayed reendothelialization, chronic inflammatory responses to metallic implants or polymer coatings, and technical challenges in navigating tortuous or heavily calcified lesions. Moreover, in patient populations with elevated bleeding risk, extensive stenting may necessitate prolonged DAPT, increasing the likelihood of adverse events.

In this scenario, drug-coated balloons (DCBs) have emerged as a promising alternative in coronary intervention (5). Unlike stents, DCBs deliver antiproliferative agents directly to the vessel wall during a brief balloon inflation without leaving behind a permanent implant (6, 7). This strategy offers several theoretical and practical advantages: preservation of vasomotion, avoidance of metal- or polymer-related hypersensitivity, reduced duration of DAPT, and the facilitation of future surgical or percutaneous interventions. The strongest evidence supporting the use of DCBs comes from their application in ISR, where they have become an established treatment option (8–10). In the *de novo* CAD setting, clinical experience has been greatest in the context of small-vessel disease, typically defined as vessels with a diameter of 2.75 mm or less (11–13). The “leave nothing behind” strategy reflects a growing preference for vascular restoration over permanent prosthesis and underpins the expanding interest in using DCBs for *de novo* lesions in large vessels, particularly in cases of greater disease complexity. The aim of this review is to summarize the potential indications for the use of DCBs in large-vessel *de novo* CAD (14–17).

## 2 DCBs technology and technical considerations

DCBs are angioplasty balloons, typically semi-compliant, that are uniformly coated with a lipophilic antiproliferative drug embedded within an excipient matrix designed to facilitate drug transfer during inflation (5, 7). Unlike DES, which provide a sustained release from a polymeric scaffold, DCBs rely on rapid drug uptake by the vessel wall during brief balloon inflation, generally lasting between 30 and 90 s (18). This necessitates a finely tuned balance between drug concentration, excipient formulation, coating morphology, and inflation technique to achieve effective inhibition of neointimal proliferation without prolonged tissue exposure or systemic toxicity. Due to substantial differences in drug formulations, excipient composition, and coating technologies, DCBs cannot be considered to have a uniform class effect, and their biological activity and clinical performance may differ significantly, even when delivering the same antiproliferative agent.

Two principal classes of drugs are currently employed in DCBs: paclitaxel, a cytotoxic agent that stabilizes microtubules and induces cell cycle arrest in the G2/M phase, and sirolimus, a cytostatic mTOR inhibitor that blocks intracellular signaling, leading to arrest at the G1 to S phase transition (Table 1) (19–22). While paclitaxel's strong tissue retention and lipophilicity make it highly compatible with balloon-mediated drug delivery, sirolimus poses greater challenges due to its reversible binding properties and lower passive diffusion into the vessel wall (23, 24). As a result, achieving sufficient tissue concentration within the limited inflation window requires enhanced delivery systems (25, 26). From an angiographic perspective, sirolimus-coated balloons (SCBs) have generally underperformed compared with paclitaxel-coated devices, while clinical outcomes have remained largely comparable (27). The assumption that sirolimus may provide a superior safety profile largely stems from earlier experience with first-generation DES, rather than from robust balloon-specific data (28, 29). In contrast, paclitaxel-coated balloons (PCBs), while effective in inhibiting neointimal hyperplasia, have been linked to adverse vascular effects such as vessel wall thinning, aneurysmal formation, and distal embolization, particularly in complex lesion subsets (30). Importantly, DCB treatment has been associated with late lumen enlargement, a phenomenon of positive vessel remodeling that leads to increased luminal area or prevention of luminal narrowing, observed in up to two-thirds of lesions treated with PCBs and one-third with SCBs (27, 31, 32).

**Table 1:** Coronary DCB platforms.

Device (company)	Dose (μg/mm^2^)	Excipient	Approval
Sirolimus			
*Magic Touch* (Concept Medical Research)	1.3	Phospholipids	CE
*Mozec* (Meril)	3	Solid lipid nanospheres	CE
*Selution* (Med Alliance)	1	Biodegradable polymer (microreservoirs)	CE
*SeQuent Please SCB* (BBraun)	4	Crystalline sirolimus	CE
*Virtue* (Caliber Therapeutics)	N/A	Biodegradable polymer (nanoparticles)	N/A
Biolimus			
*Biolimus A9* (Biosensors International)	3	Polyethylene oxide	None
Paclitaxel			
*Agent* (Boston Scientific)	2	Acetyl tributyl citrate	CE, FDA
*AngioSculpt X* (Spectranetics)	3	Nordihydroguaiaretic acid	CE
*Chocolate Touch* (QT Vascular)	3	N/A	CE
*Danubio* (Minvasys)	2.5	Butyryl-tri-hexyl citrate	CE
*Dior I and II* (Eurocor)	3	Shellac/dimethyl sulfate	CE
*Elutax SV* (Aachen Resonance)	2.2	Dextrane	CE
*Essential* (iVascular)	3	N/A	CE
*IN.PACT Falcon* (Medtronic)	3.5	Urea	CE
*Pantera Lux* (Biotronik)	3	Butyryl-tri-hexyl citrate	CE
*Prevail* (Medtronic)	3.5	Urea	CE
*Protégé* (Translumina Therapeutics)	3	Butyryl-tri-hexyl citrate	CE
*RESTORE* (Cardionovum)	3	Shellac	CE
*SeQuent Please/ NEO* (BBraun)	3	Iopromide	CE

CE, Conformité Européenne; DCB, drug-coated balloon; FDA, U.S. Food and Drug administration.

**Figure 1:**
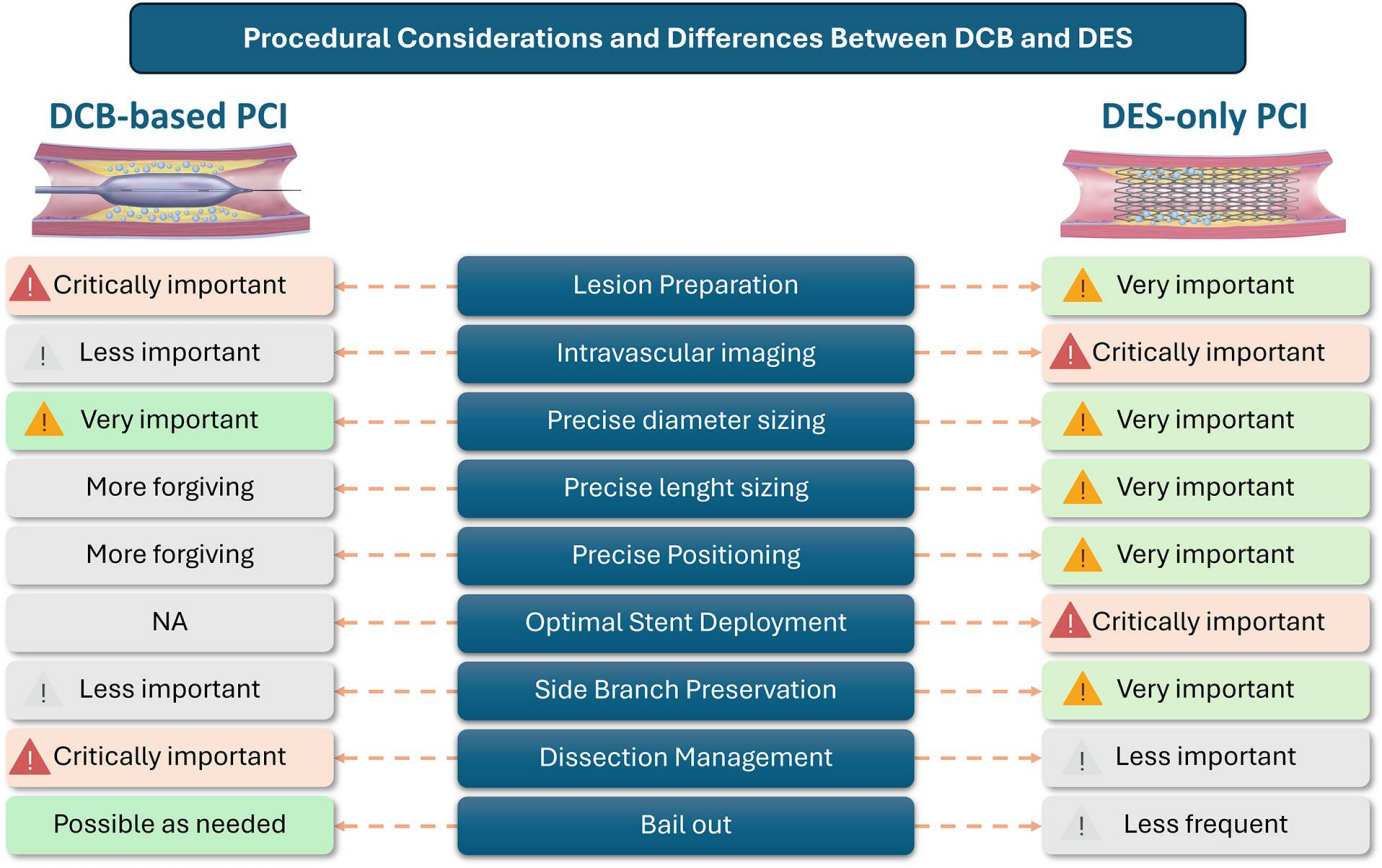
Procedural considerations and differences between DCB and DES. DES, drug-eluting stent; DCB, drug-coated balloon; PCI, percutaneous coronary intervention.

Equally critical to the success of DCB therapy is adequate lesion preparation, a procedural step that ensures optimal drug diffusion and vessel healing (Figure 1). Pre-dilation with non-compliant or specialty balloons (scoring or cutting balloons), matched in diameter to the reference vessel, is essential to reduce plaque burden and facilitate drug penetration (33). In heavily calcified or fibrotic plaques, adjunctive devices such as high-pressure balloons, rotational or orbital atherectomy, or intravascular lithotripsy may be necessary to modify the lesion and permit adequate balloon expansion (34, 35). The goal is to achieve minimal residual stenosis (<30%), Thrombolysis in Myocardial Infarction (TIMI) flow grade 3, and no significant dissection beyond type B, without persistent extraluminal contrast hang-up and with residual minimal lumen diameter (MLD) >50% of the reference vessel diameter (RVD) (36). Resting distal coronary-to-aortic pressure ratio can be of aid in decision-making in presence of advanced dissections (37).

Intravascular imaging (IVI) provides a more precise assessment of vessel size, plaque composition, and morphology, enabling optimized lesion preparation, better guidance for DCB selection, and potentially superior angiographic outcomes compared with angiography alone (38). Furthermore, controlled dissections with intimal fractures and separation from the media have been shown to facilitate positive vessel remodeling and enhance drug delivery to the vessel wall (39). Although IVI can support procedural planning and lesion optimization, evidence-based criteria to define procedural success or predict long-term clinical outcomes are still lacking (38, 40, 41). Unlike in DES implantation, where imaging is crucial for final result assessment, in DCB-PCI these studies are primarily useful for understanding lesion anatomy and guiding procedural strategy, rather than for evaluating the final treatment outcome (33).

## 3 Anatomical scenarios

Randomized data in *de novo*, non-complex lesions have so far failed to confirm the clinical equivalence of DCBs to contemporary DES (Table 2). The Real-World Evaluation of Clinical Outcomes with a Paclitaxel-Coated Balloon vs. a Sirolimus-Eluting Stent for Coronary Revascularization (REC-CAGEFREE I) trial, which enrolled 2,272 patients across 43 centers, compared paclitaxel-coated balloon (PCB) angioplasty with provisional rescue stenting (*n* = 1,133) against planned second-generation sirolimus-eluting stents (*n* = 1,139). At 24 months, the device-oriented composite endpoint [DoCE: cardiovascular death, target vessel myocardial infarction, and target lesion revascularization (TLR)] occurred in 6.4% of the DCB group vs. 3.4% of the DES group, with an absolute risk difference of 3.04% (95% CI: 1.27–4.81; *P* = 0.0008 for superiority of DES). This difference was mainly driven by a significantly higher rate of TLR in the DCB group compared with the DES group (4.4% vs. 1.3%; *P* < 0.001), whereas cardiovascular death (2.3% vs. 1.2%; *P* = 0.053) and target vessel myocardial infarction (1.9% vs. 1.6%; *P* = 0.61) occurred with similar frequency in both groups. Non-inferiority was not met, confirming DES as the standard treatment for non-complex *de novo* lesions. Indeed, in pre-specified subgroup analysis, DCBs appeared to outperform DES in large vessels (RVD ≥3.0 mm), with a higher DoCE in the DES group (7.5% vs. 2.5%; HR 3.04, 95% CI: 1.73–5.34), whereas in small vessels (RVD <3.0 mm), DoCE rates were similar between DCB and DES (5.1% vs. 4.4%; HR 1.17, 95% CI: 0.66–2.06; *p*_interaction_ = 0.020) (42).

**Table 2:** De-Novo large-vessel coronary artery disease DCBs studies.

Study	Main country/year	Experimental arm	Control arm	Sample size	Primary Endpoint	Follow-up, months	Results		*P*-value
							Experimental arm	Control arm	
Non-complex CAD									
REC-CAGEFREE trial	China/2024	DCB	DES	2,272	DoCE (CD, TV-MI, and TLR)	24	72 (6.4%)	38 (3.4%)	0.0008
Long lesions									
Gitto et al.	Italy/2023	DCB hybrid	DES only	848	TLF	24	4 (3.5%)	21 (18.2%)	0.003
Shin et al.	South Korea/2023	DCB hybrid	DES only	508	MACE (CD, MI, stroke, thrombosis, TVR, and major bleeding*)	24	10 (3.9%)	28 (11.0%)	0.002
Pan et al.	China/2023	DCB hybrid	DES only	397	TLR	24	5 (4.9%)	16 (16.3%)	0.008
Bifurcation lesions									
DCB-BIF Trial	China/2025	DCB (DES in MB)	NCB in SB (DES in MB)	784	MACE (CD, TV-MI, and TLR)	12	28 (7.2%)	49 (12.5%)	0.013
DCB-CIRCO Registry	Italy/2025	DCB in oLCx	DES in oLCx	503	TLF	24	19 (20.4%)	21 (21.3%)	0.718
BEYOND Trial	China/2020	DCB in SB (DES in MB)	POBA in SB (DES in MB)	222	TLS	9	28.7% (± 18.7%)	40.0% ± 19.0%	0.0001
PEPCAD-BIF Trial	Germany/2016	DCB in SB or dMB	POBA in SB or dMB	64	LLL	9	0.13 ± 0.31 mm	0.51 ± 0.66 mm	0.013
BABILON Trial	Spain/2014	DCB in SB + BMS in MB	DES in MB	108	LLL	9	−0.03 ± 0.51 mm	0.04 ± 0.76 mm	0.983
Herrador et al.	Spain/2013	DCB in SB (DES in MB)	POBA in SB (DES in MB)	100	Binary restenosis	12	6 (15%)	12 (27%)	0.15
CTOs									
Shin et al.	South Korea/2024	DCB hybrid	DES only	861	MACE (CD, MI, thrombosis, TLR, and major bleeding*)	24	5 (3.1%)	74 (13.2%)	0.001
PEPCAD-CTO study	Germany/2012	DCB + BMS	DES	96	LLL	6	0.64 ± 0.69 mm	0.43 ± 0.64 mm	0.14
Severely calcified lesions									
Mitsui et al.	Japan/2023	OA + DCB	OA + DES	135	MACE (CD, MI, and TLR)	12	4 (9.7%)	3 (3.4%)	0.134
Dong et al.	China/2023	RA + DCB	RA + DES	318	MACE (death, MI, TLR, and stroke)	12	7 (12.3%)	49 (18.8%)	0.25
Iwasaki et al.	Japan/2020	RA + DCB	RA + DES	165	MACE (death, TV-MI, TLR, and major bleeding*)	12	7 (11%)	7 (8%)	0.30
Ueno et al.	Japan/2019	RA + DCB	RA + DES	123	TLR	36	11 (15.6%)	16 (16.3%)	0.99
Thrombotic lesions									
STEMI									
REVELATION trial	Netherlands/2019	DCB	DES	120	FFR	9	0.92 ± 0.05 mm	0.91 ± 0.06 mm	0.27
Gobić et al.	Croatia/2017	DCB	DES	75	LLL	6	−0.09 ± 0.09 mm	0.10 ± 0.19 mm	<0.05
DEB-AMI trial	Netherlands/2015	DCB	BMS DCB + BMS DES	190	LLL	6	0.51 ± 0.59 mm	0.74 ± 0.57 mm 0.64 ± 0.56 mm 0.21 ± 0.32 mm	0.44 0.88 <0.01
NSTEMI									
PEPCAD NSTEMI trial	Germany/2020	DCB	BMS or DES	210	TLF	9	4 (3.8%)	7 (6.6%)	0.53
High bleeding risk									
REC-CAGEFREE II Trial	China/2025	DAPT descalation	Standard DAPT	1,948	MACE (death, stroke, MI, revascularisation, and major bleeding*)	12	87 (8.9%)	84 (8.6%)	0.013
EASTBOURNE registry	Italy/2025	SAPT	DAPT	2,123	TLR	12	7.7%	5.6%	0.68
Diabetes									
EASTBOURNE DIABETES registry	Italy/2024	Diabetic	Non-diabetic patients	2,123	TLR	12	6.5%	4.7%	NS

BMS, bare metal stent; CAC, coronary artery calcification; CAD, coronary artery disease; CD, cardiovascular death; CTO, chronic total occlusion; DAPT, dual antiplatelet therapy; DES, drug-eluting stent; DCB, drug-coated balloon; DoCE, device-oriented composite endpoint; dMB, distal main branch; FFR, fractional flow reserve; LLL, late lumen loss; MACE, major adverse cardiac event; MI, myocardial infarction; MB, main branch; NCB, non-compliant balloon; NS, not significant; NSTEMI, non-ST-segment elevation myocardial infarction; OA, orbital atherectomy; oLCx, ostial left circumflex; PSM, propensity score matching; RA, rotational atherectomy; RCT, randomized controlled trial; SAPT, single antiplatelet therapy; SB, side branch; STEMI, ST-segment elevation myocardial infarction; TLF, target lesion failure; TLR, target lesion revascularization; TV-MI, target vessel myocardial infarction.

* Bleeding academic research consortium bleeding type 3 or greater.

### 3.1 Long coronary lesions

The treatment of long coronary lesions remains one of the most demanding scenarios in PCI, frequently requiring multiple overlapping DES, creating a “full metal jacket” configuration that is associated with higher risks of restenosis, stent thrombosis, and impaired vascular remodeling (1, 2, 4).

**Figure 2:**
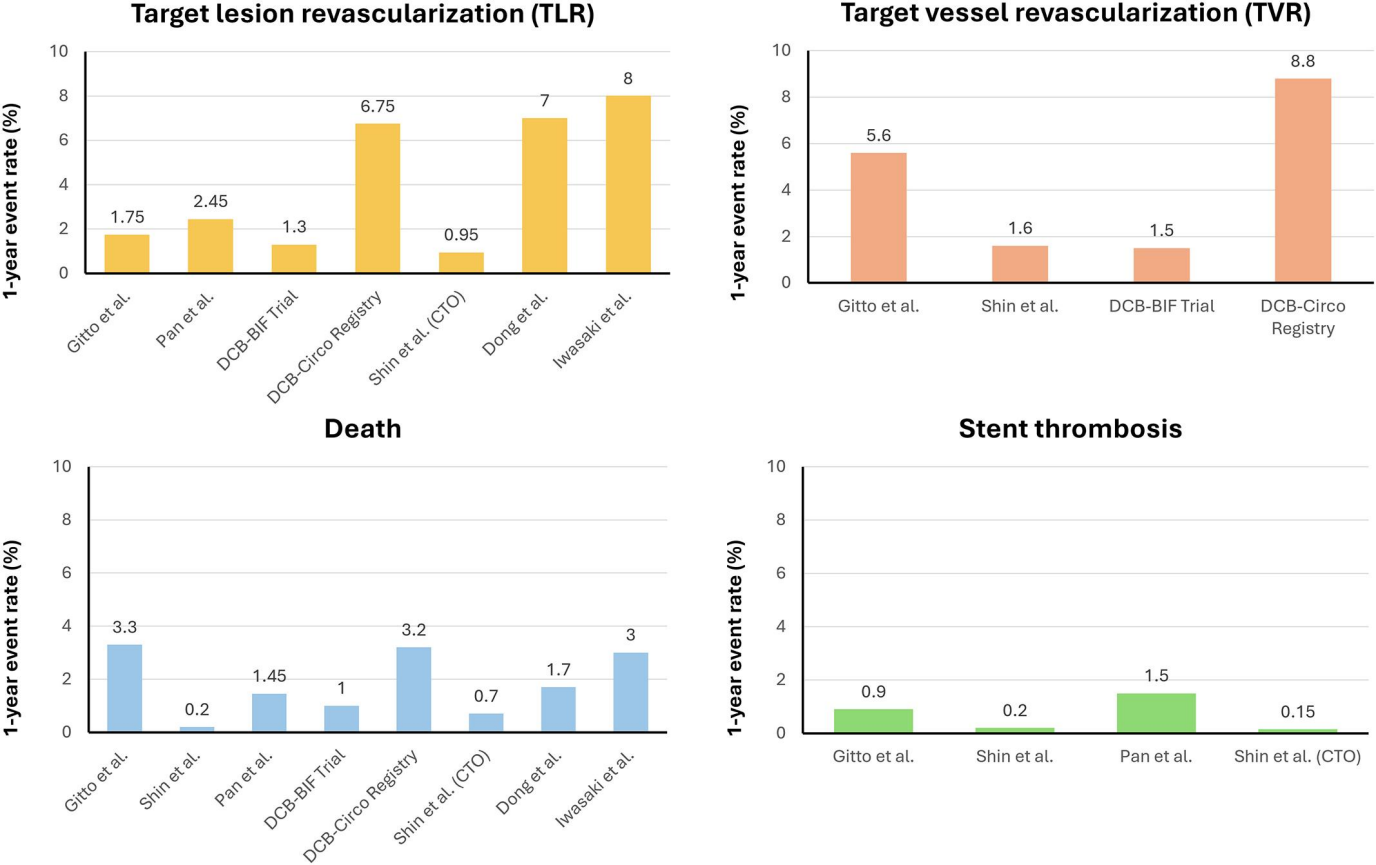
Clinical and angiographic outcomes at 1-year follow-up. CTO, chronic total occlusion.

By contrast, growing evidence supports the role of DCBs in complex or long lesions. In a multicenter observational study, Shin et al. evaluated 254 patients with multivessel CAD treated with a DCB-based approach (34.3% DCB-only, 65.7% hybrid DES + DCB) compared with a propensity-matched DES-only cohort. At 2 years, the DCB-based group exhibited a significantly lower incidence of major adverse cardiovascular events (MACE: 3.9% vs. 11.0%; *P* = 0.002), driven by reductions in cardiac death and major bleeding. Importantly, the DCB-based strategy reduced the number and cumulative length of implanted stents by approximately 65% (43) (Figure 2).

Similarly, a PS-matched study from our group analyzed patients with long LAD lesions (mean treated length 65 mm in the DCB group vs. 56 mm in the DES group), showing a reduced risk for target lesion revascularization (TLR) and target lesion failure (TLF) at 2 years in the DCB cohort [hazard ratio (HR) 0.20, 95% CI: 0.07–0.58; *P* =  0.003]. Notably, most TLR events in the DCB group clustered within the first 6 months, after which event rates plateaued, whereas the DES cohort exhibited a steady increase in events over time, reflecting progressive stent-related adverse remodeling (44).

In a similar PS-matched analysis, Pan et al. focused on ostial LAD lesions, reporting a TLR rate of 4.9% in the DCB group compared with 16.3% in the DES group, and a lower incidence of MACE (7.8% vs. 19.3%). Moreover, late lumen loss (LLL) was significantly reduced after DCB-based therapy (−0.02 ± 0.57 mm) compared with DES (0.30 ± 0.27 mm; *P* < 0.001), suggesting sustained vessel patency despite lower acute lumen gain (45).

### 3.2 Bifurcation lesions

Coronary bifurcation lesions account for approximately 15%–20% of all PCIs and remain a complex subset due to the inherent risk of side branch (SB) compromise during main branch (MB) stenting. The European Bifurcation Club recommends a provisional stenting strategy in most cases, with SB balloon dilatation or stenting performed only if necessary (46). Nevertheless, MB stenting can alter bifurcation geometry, reduce SB flow, and increase the risk of ischemia, highlighting the potential role of DCBs in preserving SB patency.

Randomized trials provide evidence for the efficacy of DCBs in the SB. In the DCB-BIF trial, 784 patients with true bifurcation lesions and SB diameter stenosis ≥70% after MB stenting were randomized to paclitaxel-coated balloons (*n* = 391) or non-compliant balloons (*n* = 393). At 1-year follow-up, major adverse cardiac events (MACE) occurred in 7.2% of the DCB group vs. 12.5% in the control group (HR 0.56; 95% CI: 0.35–0.88; *P* = 0.013), driven primarily by a reduction in myocardial infarction. Procedural success and crossover to two-stent strategies were similar in both arms (47).

Similarly, the PEPCAD-BIF trial enrolled 64 patients with non-left main bifurcations, randomized to DCB or plain balloon angioplasty (POBA). At 9 months, LLL was 0.13 mm in the DCB group vs. 0.51 mm in the POBA group (*P* = 0.013), restenosis occurred in 6% vs. 26%, and TLR was required in 1 vs. 3 patients, respectively. The BEYOND trial included 222 patients and demonstrated that target lesion stenosis at 9 months was 28.7% ± 18.7% with DCB vs. 40.0% ± 19.0% with conventional balloons (Δ = −11.3%; *P* < 0.0001), with LLL of −0.06 ± 0.32 mm vs. 0.18 ± 0.34 mm (*P* < 0.0001), and no significant difference in MACE (0.9% vs. 3.7%; *P* = 0.16) (48).

The BABILON trial evaluated paclitaxel-coated balloons (PCBs) in 108 patients with *de novo* bifurcation lesions, randomized to either sequential MB/SB predilatation with PCB plus BMS implantation in the MB (SB treated only with PCB unless stenting was required) or standard MB/SB predilatation with DES in the MB, with provisional T-stenting of the SB if needed. At 9-month, LLL in the MB was 0.31 ± 0.48 mm in the PCB/BMS group vs. 0.16 ± 0.38 mm in the DES group, with a mean difference of 0.15 mm, confirming non-inferiority. In the SB, LLL was minimal and comparable between the two groups (−0.04 ± 0.76 mm vs. −0.03 ± 0.51 mm). However, MACE occurred more frequently in the PCB group (17.3% vs. 7.1%), as did TLR (15.4% vs. 3.6%), largely due to higher MB restenosis (13.5% vs. 1.8%). Although overall outdated due to the use of BMS in the MB, these findings indicate that PCB are effective in preserving SB patency and bifurcation geometry (49).

Observational studies in more complex bifurcations corroborate the benefit of DCBs. In a multicenter study of 152 patients undergoing PCI for ostial left circumflex lesions using paclitaxel- or sirolimus-coated balloons, 2-year TLF was 19.0% vs. 19.8% in a DES-treated historical cohort (HR 1.05; 95% CI: 0.64–1.75), despite longer lesion lengths (25.5 mm vs. 18.0 mm; *P* < 0.001) and reduced fluoroscopy time (31.0 vs. 20.0 min) and contrast volume (200 vs. 150 mL) in the DCB group (50). Another study comparing DEB to conventional balloon angioplasty in 50 patients per group undergoing provisional T stenting demonstrated lower LLL (0.09 mm vs. 0.40 mm; *P* = 0.01), reduced restenosis (7% vs. 20%), and fewer MACE (11% vs. 24%) in the DCB group at 12-month follow-up (51).

Taken together, these data indicate that DCBs provide effective SB treatment in bifurcation lesions, reducing LLL, restenosis, and TLR while preserving bifurcation geometry as compared to DES. However, despite encouraging results, the European Bifurcation Club does not currently recommend routine DCB use for *de novo* bifurcation lesions due to the limited number of comparative studies against DES (46). Furthermore, evidence supporting the use of DCBs in left main (LM) bifurcation lesions remains scarce (52).

### 3.3 Chronic total occlusions

Chronic total occlusions (CTOs) represent one of the most challenging subsets of coronary lesions for PCI, due to their dense fibrotic or calcific nature, frequent underestimation of vessel size, and higher risk of stent under-expansion or malapposition (53). These factors contribute to increased rates of restenosis, stent thrombosis, and adverse cardiac events when conventional BMS or DES are used (54).

Recent multicenter observational studies suggest that DCB-based strategies, either alone or combined with DES, can achieve favorable outcomes in CTOs. In a cohort of 200 patients treated with DCB-based PCI, 49% received DCB alone, while 51% underwent a hybrid approach combining DCB with DES. Bailout stenting was required in only 3.5% of cases. Compared with 661 patients receiving DES-only CTO interventions, the DCB-based group had significantly fewer stents per patient (median 1.0 vs. 2.0), shorter total stent lengths (median 6.5 mm vs. 42 mm), and reduced use of small-diameter stents ≤2.5 mm (9.8% vs. 36.5%). At 2-year follow-up, MACEs occurred in 3.1% of the DCB group vs. 13.2% of the DES-only group, reflecting the clinical benefit of reducing stent burden (55).

While randomized evidence remains limited, available data support a hybrid strategy in which DCBs are used to treat the diffuse lesions, complemented by DES in those segments in which scaffolding is necessary. This approach appears to preserve long-term vessel patency and reduce stent-related complications, offering a promising alternative to conventional stenting in CTO interventions. Conversely, the potential advantages of DCB inflation within the subintimal space, particularly in the setting of an “investment” procedure, warrant further investigation.

### 3.4 Severely calcified lesions

Coronary artery calcification (CAC) continues to represent a major procedural challenge in contemporary PCI, even in the era of new-generation DES (56, 57). Calcified lesions are associated with higher rates of restenosis, stent thrombosis, and adverse cardiovascular events, largely due to difficulties in achieving adequate predilation, suboptimal stent expansion, and irregular plaque morphology (58, 59). Consequently, lesion preparation with adjunctive devices such as rotational atherectomy (RA) and orbital atherectomy (OA), is often necessary to optimize outcomes prior to stent implantation.

DCBs have emerged as a promising alternative strategy for CAC, particularly when combined with lesion-modifying techniques, since drug delivery may be hindered by extensive calcification. Several recent studies have investigated DCB outcomes in calcified lesions following RA or OA. In a single-center analysis of 194 lesions treated with RA, subsequent DCB therapy demonstrated comparable 1-year MACE rates to DES (11% vs. 8%, *P* = 0.30), with similar rates of target lesion revascularization (8% vs. 4%) and negligible differences in cardiac or noncardiac mortality (1 cardiac and 2 noncardiac deaths for each group) (60). Similarly, in patients undergoing OA, 1-year MACE occurred in 9.7% after DCB and 3.4% after DES (*P* = 0.136), despite thicker calcium plaques and smaller postprocedural lumen areas in the DCB group, suggesting feasibility when adequate lesion preparation is achieved (61).

Long-term data from an intracoronary imaging-guided RA cohort (123 patients, 166 lesions) showed that target lesion revascularization and target vessel revascularization at three years were comparable between DCB and DES (15.6% vs. 16.3% and 15.6% vs. 23.3%, respectively), while late lumen loss was significantly lower with DCB (0.09 mm vs. 0.52 mm, *P* = 0.009) (62). Moreover, a retrospective study assessed 318 patients with severe CAC undergoing RA-assisted PCI, including 57 patients treated with DCB and 261 with DES. Despite more complex anatomy in the RA/DES group, including left main, bifurcation, and multivessel disease, rates of TLR (13.8% vs. 7.0%) and MACE (18.8% vs. 12.3%) did not differ significantly between groups (*P* > 0.05) (63).

A meta-analysis encompassing 1,141 patients and 1,176 calcified coronary lesions across five studies confirmed that DCBs are comparable to DES in terms of 12-month MACE (Risk Ratio = 0.86, 95% CI: 0.62–1.19, *P* = 0.36), cardiac death (RR = 0.59, 95% CI: 0.23–1.53, *P* = 0.28), myocardial infarction (RR = 0.89, 95% CI: 0.25–3.24, *P* = 0.87), and TLR (RR = 1.10, 95% CI: 0.68–1.77, *P* = 0.70). While acute angiographic results were lower with DCBs, including an average acute gain reduction of 0.65 mm and a minimal lumen diameter 0.75 mm smaller than with DES, follow-up at 12 months demonstrated improved LLL (−0.34 mm), suggesting a durable effect of the stent-less strategy (64).

However, randomized data on the use of DCBs in severely calcified coronary lesions are still lacking. Moreover, the presence of extensive calcium may limit drug absorption, potentially reducing antiproliferative efficacy and contributing to higher rates of restenosis in inadequately prepared lesions.

### 3.5 Thrombotic lesions

Acute coronary syndromes (ACS) present unique challenges for PCI, as high thrombus burden, vessel spasm, and microvascular obstruction can increase the risk of distal embolization, no-reflow, and stent malapposition. In this context, DCBs have been investigated as an alternative or complement to conventional stenting. Although residual thrombus may interfere with homogeneous drug delivery to the vessel wall and reduce antiproliferative efficacy, the risk of stent undersizing in ACS further reinforces the rationale for a DCB-based strategy, potentially avoiding the complications of malapposition and suboptimal stent expansion (65). Indeed, stent-related complications remain a concern, with cardiac adverse events (including death, MI, or TLR) reported in up to 32.4% of patients at 10-year follow-up (66).

In the STEMI setting, the REVELATION trial randomized 120 patients with non-severely calcified culprit lesions to DCB or DES. At 9 months, mean fractional flow reserve (FFR) was 0.92 ± 0.05 in the DCB group and 0.91 ± 0.06 in the DES group, with only one abrupt vessel closure in the DCB arm and two patients requiring non-urgent target lesion revascularization overall (67). The DEB-AMI trial assessed 150 STEMI patients divided into three groups (BMS, DCB with BMS, and DES) reporting a LLL of 0.74 ± 0.57 mm, 0.64 ± 0.56 mm, and 0.21 ± 0.32 mm, respectively. Corresponding binary restenosis rates were 26.2%, 28.6%, and 4.7%, and MACE occurred in 23.5%, 20.0%, and 4.1% of patients, respectively (68). Gobić et al. further explored DCB-only therapy in STEMI in a feasibility study of 75 patients, randomized to DCB (*n* = 38) or DES (*n* = 37). Reinfarction occurred in 5.3% of DCB patients vs. 5.4% in the DES group after 1 month. At six months, no major adverse cardiac events were reported in the DCB group, compared with 5.4% in the DES group. Late lumen loss was notably lower in the DCB arm (−0.09 ± 0.09 mm) compared with the DES group (0.10 ± 0.19 mm, *P* < 0.05), supporting the safety and feasibility of DCB-only strategies in primary PCI (69).

In NSTEMI, the PEPCAD NSTEMI trial enrolled 210 patients and demonstrated a TLF rate of 3.8% in the DCB group vs. 6.6% in the stent group over an average follow-up of 9.2 months. MACE occurred in 6.7% of DCB-treated patients vs. 14.2% in the stent-treated cohort, with similar results observed in per-protocol analyses. Most patients (85%) received DCB alone, with only 15% requiring additional stenting (70).

Meta-analytic data pooling 497 patients across three randomized trials and one observational study showed comparable rates of MACE (5% vs. 4.4%), all-cause mortality (0.02% vs. 0.04%), cardiac death (0.01% vs. 0.02%), myocardial infarction (0% vs. 1.4%), and TLR (3.7% vs. 2%) between DCB and DES in AMI patients. Late lumen loss was also similar (mean difference 0.04 mm). These findings reinforce that DCB treatment can provide effective vessel patency and clinical safety, particularly in lesions with minimal thrombus burden (71).

Overall, current evidence suggests that DCB therapy in ACS is a safe and viable alternative to conventional stenting in carefully selected patients, offering the advantage of reduced stent implantation while preserving favorable angiographic and clinical outcomes.

## 4 Clinical scenarios

A DCB-based PCI approach might become a viable treatment option for diabetic patients, often presenting complex and recalcitrant CAD, and for high-bleeding risk (HBR) patients requiring shortened dual antiplatelet therapy (DAPT) durations.

### 4.1 High bleeding risk patients

Management of patients at high bleeding risk (HBR) undergoing PCI remains a major clinical challenge, given the delicate balance between ischemic protection and avoidance of hemorrhagic complications. In this context, DCB angioplasty has attracted considerable interest as the absence of a permanent metallic implant might potentially allow for more flexible antithrombotic strategies (72). Expert consensus documents currently recommend a shortened course of DAPT, often limited to 4 weeks after DCB-only PCI for patients presenting with chronic coronary syndromes, with the possibility of reducing the duration to as little as 2 weeks in cases of very high bleeding risk (5). Retrospective analyses and small randomized trials have recently suggested that DCB-only PCI can be performed safely with abbreviated DAPT regimens, and in some instances even with single antiplatelet therapy (SAPT) (73, 74). Unlike contemporary DES, for which short DAPT regimens are supported by large randomized trials, robust evidence for abbreviated DAPT following DCB, particularly in thrombotic lesions, remains limited (75).

In a retrospective cohort analyzed by Räsänen et al., SAPT following DCB PCI was associated with low rates of adverse events, with a 1-year incidence of MACE at 4.7% and cardiovascular death at 2.9% (74). Similarly, Cortese et al. observed no significant differences between SAPT and DAPT in terms of MACE or its components, while noting fewer bleeding complications in patients treated with SAPT (76). Two large-scale prospective datasets have recently provided further insights. The REC-CAGEFREE II trial, a multicenter randomized study of 1,948 East Asian ACS patients undergoing DCB-only angioplasty, directly compared a stepwise DAPT de-escalation regimen (one month of aspirin plus ticagrelor, followed by five months of ticagrelor monotherapy and then aspirin alone) with standard 12-month DAPT. The primary endpoint of net adverse clinical events at 12 months occurred in 8.9% of patients in the de-escalation group vs. 8.6% in the standard DAPT group, meeting the criteria for non-inferiority. Importantly, the incidence of major bleeding (BARC 3–5) was significantly reduced in the de-escalation group (0.4% vs. 1.6%, *P* = 0.008), suggesting that a less intensive approach is both safe and effective in this setting (77).

In the EASTBOURNE registry, which enrolled over 2,100 patients treated with sirolimus-coated balloons, among 113 patients receiving SAPT, no cases of acute vessel occlusion or early target lesion revascularization were observed. At 12 months, the rate of TLR was similar between SAPT and DAPT groups (7.7% vs. 5.6%, *P* = 0.6), while cumulative MACE rates also showed no significant difference (11.2% vs. 8.9%, *P* = 0.4). These findings support the safety of SAPT even in complex cohorts, with the potential to mitigate bleeding risks without compromising ischemic protection (78).

### 4.2 Diabetes Mellitus

Diabetes mellitus (DM) poses a significant challenge for PCI, as affected patients often exhibit diffuse, multivessel disease, longer lesions of smaller diameter, and an elevated risk of restenosis, stent thrombosis, and recurrent myocardial infarction following conventional stent implantation (79–81). Consequently, a stent-free strategy using DCBs has emerged as an appealing alternative in this high-risk population.

A recent meta-analysis pooling six randomized studies and 847 patients with small-vessel disease provided important insights into the comparative efficacy of DCB vs. DES in diabetic patients. The analysis demonstrated a consistent reduction in MACE with DCB (RR: 0.60, 95% CI: 0.39–0.93), as well as a lower risk of myocardial infarction (RR: 0.42, 95% CI: 0.19–0.94) and target lesion revascularization (RR: 0.24, 95% CI: 0.08–0.69). Other favorable outcomes included reduced target vessel revascularization (RR: 0.33, 95% CI: 0.18–0.63), binary restenosis (RR: 0.27, 95% CI: 0.11–0.68), and late lumen loss (mean difference −0.31 mm, 95% CI: −0.36 to −0.27). By contrast, technical success, all-cause mortality, minimal lumen diameter, and net lumen gain were comparable between the two strategies, suggesting that the short-term angiographic and clinical performance of DCB is at least equivalent, and in some respects superior, to DES in this high-risk population (82).

In the EASTBOURNE study, diabetic patients (*N* = 864) had numerically higher rates of target lesion revascularization at 1 year compared to non-diabetics (6.5% vs. 4.7%, HR 1.38, 95% CI: 0.91–2.08), as well as all-cause death (3.8% vs. 2.6%, HR 1.81, 95% CI: 0.95–3.46) and MACE (12.2% vs. 8.9%, HR 1.26, 95% CI: 0.92–1.74). Importantly, the incidence of spontaneous myocardial infarction was significantly higher among diabetic patients (3.4% vs. 1.5%, HR 2.15, 95% CI: 1.09–4.25), while bleeding complications did not differ. Stratified analyses indicated that SCB outcomes were similar in de-novo lesions and in-stent restenosis, and that diabetic status did not significantly affect device performance, with angiographic success exceeding 97% in both groups (83).

Overall, current evidence indicates that DCB angioplasty is feasible in diabetic patients and may reduce restenosis and repeat revascularization compared with DES, potentially mitigating their usual prognostic disadvantage. However, higher event rates observed in registries highlight the need for further randomized studies to confirm long-term safety and efficacy.

## 5 Future perspectives

The expanding body of evidence on DCBs in complex coronary interventions highlights both their promise and the need for continued research. Future directions are likely to focus on optimizing patient and lesion selection, refining procedural techniques, and expanding indications beyond current practice. Advanced intracoronary imaging and physiological assessments may improve lesion preparation and procedural success, particularly in challenging subsets such as severely calcified lesions, long diffuse disease, bifurcations, and CTOs.

Technological innovations in DCB design, including enhanced drug formulations, excipients, and balloon delivery systems, may improve drug uptake and reduce variability in vessel response. To date, no randomized studies have directly compared sirolimus- vs. paclitaxel-coated balloons, and such trials will be critical in identifying which technology may be superior in specific clinical scenarios (84).

**Figure 3:**
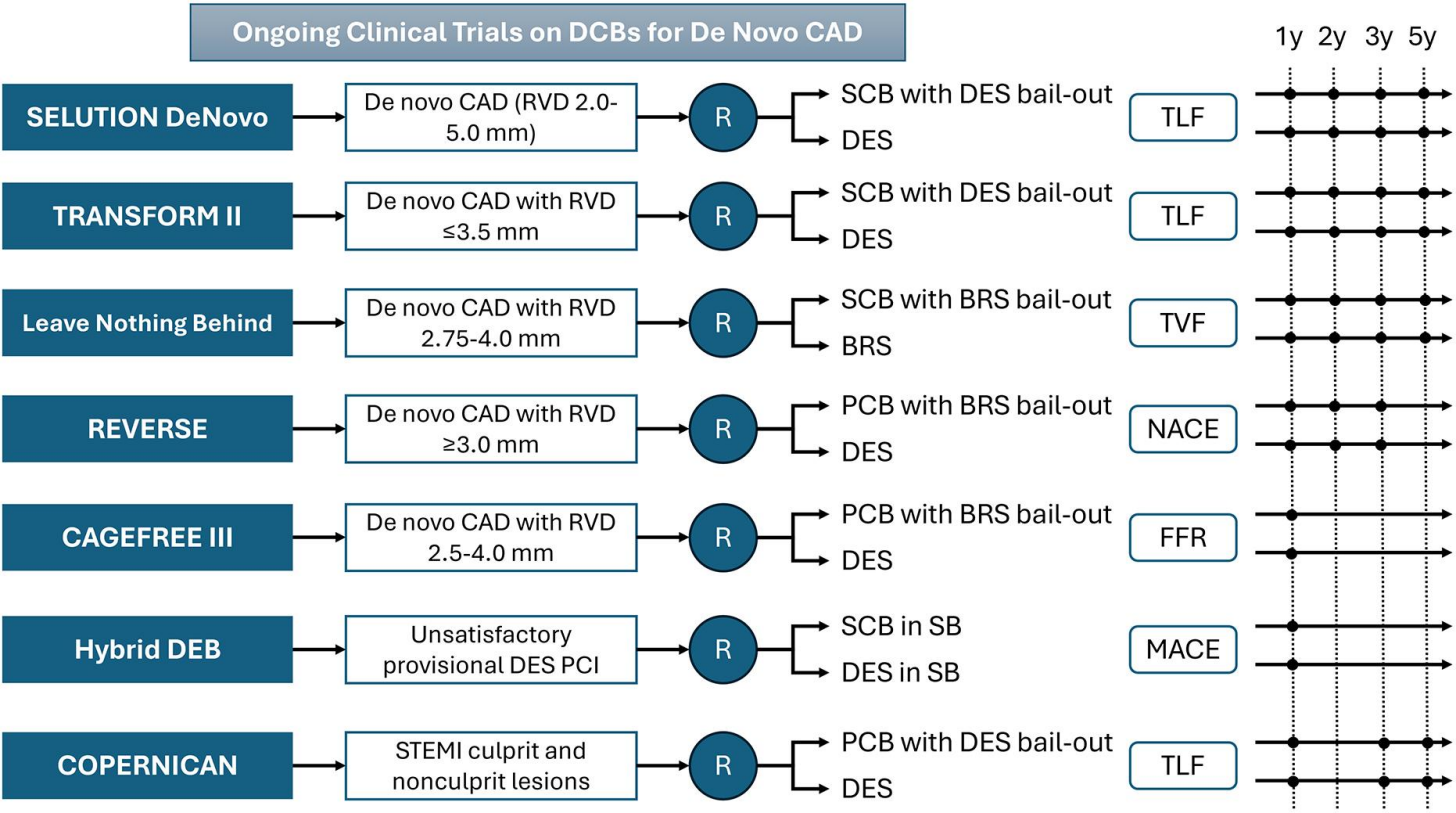
Ongoing clinical trials on DCBs for *de novo* large-vessel CAD. BRS, bioresorbable scaffold; CAD, coronary artery disease; DES, drug-eluting stent; DCB, drug-coated balloon; FFR, fractional flow reserve; LLL, late lumen loss; MACE, major adverse cardiac event; MB, main branch; NACE, net adverse cardiac events; PCI, percutaneous coronary intervention; RVD, reference vessel diameter; SB, side branch; TLF, target lesion failure; TVF, target vessel failure.

Ultimately, further large-scale, multicenter randomized trials powered for clinical endpoints will help determine whether a “leave nothing behind” approach can consistently improve outcomes compared with a universal DES implantation strategy, ultimately refining clinical guidelines and expanding the role of DCBs in everyday practice (Table 3 and Figure 3). In this context, the recently presented 1-year results of the SELUTION *de novo* trial are encouraging. A SCB strategy with provisional stenting was noninferior to upfront DES implantation for the treatment of *de novo* coronary lesions—predominantly located in large vessels and meeting predefined complexity criteria. TVF occurred in 5.3% of patients in the DCB group vs. 4.4% in the DES group (*P* = 0.02 for noninferiority), with comparable rates of cardiac death (0.7% vs. 1.0%), target-vessel MI (2.7% vs. 2.6%), and lesion thrombosis (0.1% vs. 0.3%).

**Table 3:** Ongoing DCB studies.

Study Title	Design	DCB	Primary endpoint	RVD	Target sample size	Follow-up, months
De-novo large-vessel CAD						
SELUTION DeNovo—A Prospective Randomized, Multi-center, International, Single-blind, Clinical Trial Compared the Selution DCB Strategy vs. DES Strategy *(Clinicaltrials.gov NCT04859985)*	Prospective, multicenter, singleblind, randomized clinical trial (DCB vs. DES)	Selution SLR (M.A. Med Alliance S.A.)	TLF	2.0–5.0 mm	3,326	12
Sirolimus-coated Balloon vs. Drugeluting Stent in Native Coronary Vessels (TRANSFORM II) *(Clinicaltrials.gov NCT04893291)*	Prospective, multicentric, openlabel, randomized clinical trial (DCB vs. DES)	MagicTouch (Surat, India)	TLF	≤3.5 mm	1,820	Up to 60
Leave Nothing Behind Study Which Compares DCB With Bail Out BRS vs. BRS Strategy Alone (LNB) *(Clinicaltrials.gov NCT07038408)*	Prospective, multicentric, openlabel, randomized clinical trial (DCB with BRS bailout vs. BRS)	Mozec SEB (Meril Life Sciences, Vapi, India)	TVF	2.75–4.0 mm	2,256	12
Drug-Coated Balloon vs. Drug-Eluting Stent for Clinical Outcomes in Patients With Large Coronary Artery Disease (REVERSE) *(Clinicaltrials.gov NCT05846893)*	Prospective, randomized, openlabel, international multicenter trial (DCB vs. DES)	SeQuent Please NEO (Braun, Melsungen, Berlin, Germany)	NACE	≥3.0 mm	1,436	12
A Trial of DCB vs DES in the Treatment of *de novo*Large Diameter Coronary Atherosclerotic Stenosis (LARGE ONE) *(Clinicaltrials.gov NCT05961787)*	Prospective, Multicenter, Randomized Controlled Trial (DCB vs. DES)	SeQuent Please (Braun, Melsungen, Berlin, Germany)	LLL	3.0–4.0 mm	134	13
Fractional Flow Reserve Guided Drug Coated Balloon Only Strategy in *de novo*coronary Lesions (FADDY) *(Clinicaltrials.gov NCT03452904)*	Prospective, randomized clinical trial (DCB vs. DES)	Any	FFR	2.5–3.5 mm	80	9
Paclitaxel-Coated Balloon vs. Zotarolimus-Eluting Stent for Treatment of *de novo* Coronary Artery Lesions (CAGEFREE III) *(Clinicaltrials.gov NCT05209412)*	Prospective, open-label, randomized clinical trial (DCB vs. DES)	VesselinR (Lepu Medical Technology, Changping Qu, China)	FFR	2.5–4.0 mm	370	12
Drug-coated Balloons in Big *de novo* Coronary Disease (DCB-LVD) *(Clinicaltrials.gov NCT05550233)*	Prospective, multicenter, openlabel, randomized, non-inferiority clinical trial (DCB vs. DES)	Any	LLL	≥3.0 mm	240	12
Diffuse CAD						
Drug-Coated Balloon in Combination with New Generation Drug-Eluting Stent for *de novo* Diffuse Disease Treatment (HYPER) *(Clinicaltrials.gov NCT03939468)*	Prospective, nonrandomized, single-arm, multicenter study (DCB + DES)	Restore DCB (Cardionovum, Bonn, Germany)	DoCE	>2.75 mm for DES and 2–2.75 mm for DCB	100	12
Comparison of Safety and Efficacy of Coronary Drug-coated Balloon (DCB) Combined with Spot Stenting of Drugeluting Stent (DES) vs. Secondgeneration DES for Treating Diffuse Coronary Artery Lesion *(Clinicaltrials.gov NCT03817801)*	Prospective, randomized clinical trial (DCB + DES vs. DES)	SeQuent Please (Braun, Melsungen, Berlin, Germany)	LLL	2.5–4.0 mm	140	36
Bifurcation lesions						
Bifurcation PCI With a Hybrid Strategy With Drug Eluting Balloons vs. a Stepwise Provisional Two-stent Strategy (Hybrid DEB) *(Clinicaltrials.gov NCT05731687)*	Prospective, randomized controlled, single blinded, multicenter trial (DCB vs. DES)	MagicTouch (Surat, India)	MACE	Bifurcation lesion after provisional DES PCI	500	12
Clinical Trial on Safety and Efficacy of Drug-coated Balloon in Treatment of Coronary Bifurcation Lesions (BJDCB-BIF) *(Clinicaltrials.gov NCT03223974)*	Prospective, randomized clinical trial (DCB vs. DES)	Any	LLL	Bifurcation lesions	80	24
Bingo Drug-eluting Balloon vs. a Drugeluting Stent for Coronary Bifurcation Lesions *(Clinicaltrials.gov NCT06441539)*	Prospective, open-label multicenter, randomized, noninferiority clinical trial (DCB vs. DES)	Bingo (Yinyi Liaoning Biotech Co., Ltd.)	LLL in MB	Bifurcation lesions	218	9
Thrombotic lesions						
Drug-coated Balloon vs. Drug-eluting Stent in the Treatment of Coronary Artery Lesions in STEMI Patients in *de novo* Coronary Lesions *(Clinicaltrials.gov NCT04072081)*	Prospective, multicenter, randomized clinical trial (DCB vs. DES)	Any	LLL	2.5–3.5 mm	4,000	24
Reduced stent strategy vs. conventional percutaneous coronary revascularization in patients presenting with STEMI (COPERNICAN) *(Clinicaltrials.gov NCT06353594)*	Prospective, multicenter, randomized, noninferiority clinical trial	Any PCB	TLF	Culprit and nonculprit lesions	1,272	Up to 120
High bleeding risk						
Comparison of Scoring Balloon and Conventional Balloon Predilation Before Drug Coated Balloon for *de novo* Lesion in Patients with High Bleeding Risk (PREPARE-NSE) *(Clinicaltrials.gov NCT03817801)*	Prospective, randomized clinical trial (DCB with scoring balloon dilation vs. DCB with standard balloon dilation)	Any	FFR	*De novo* CAD in patients with HBR	60	6

BARC, bleeding academic research consortium; BMS, bare metal stent; BRS, bioresorbable scaffold; CAD, coronary artery disease; DES, drug-eluting stent; DCB, drug-coated balloon; DoCE, device oriented composite outcome; DS, diameter stenosis; FFR, fractional flow reserve; HBR, high bleeding risk; LLL, late lumen loss; MACE, major adverse cardiac event; MB, main branch; MLD, minimum lumen diameter; NACE, net adverse cardiac events; PCB, paclitaxel-coated balloon; PCI, percutaneous coronary intervention; RVD, reference vessel diameter; STEMI, ST-segment elevation myocardial infarction; TLF, target lesion failure; TVF, target vessel failure.

**CENTRAL ILLUSTRATION:**
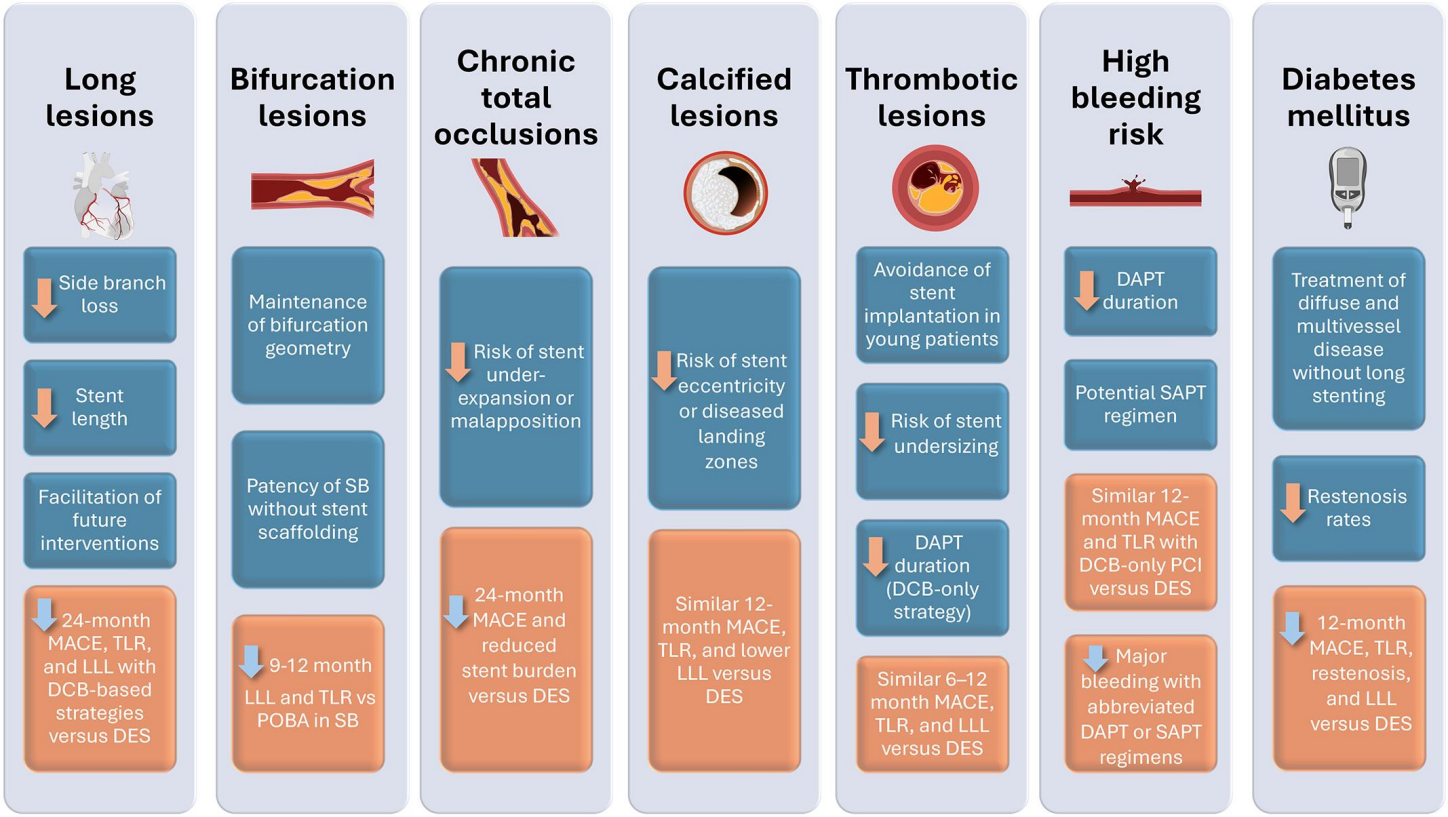
Drug-Coated Balloons in Complex Large-Vessel Coronary Artery Disease: A Comprehensive Review of Current Evidence and Future Perspectives. DAPT, dual antiplatelet therapy; DES, drug-eluting stent; DCB, drug-coated balloon; LLL, late lumen loss; MACE, major adverse cardiac event; PCI, percutaneous coronary intervention; POBA, plain old balloon angioplasty; SAPT, single antiplatelet therapy; SB, side branch; TLF, target lesion failure; TLR, target lesion revascularization.

## 6 Conclusions

DCBs represent a promising alternative to DES in a variety of complex coronary lesions, offering the advantages of reduced stent burden, preserved vessel physiology, and lower rates of late lumen loss and restenosis (Central Illustration). Future large-scale randomized trials are essential to confirm these findings, refine patient selection, and define the long-term role of DCBs in contemporary PCI.
